# Die Another Way: Interplay between Influenza A Virus, Inflammation and Cell Death

**DOI:** 10.3390/v12040401

**Published:** 2020-04-04

**Authors:** Gabriel Laghlali, Kate E. Lawlor, Michelle D. Tate

**Affiliations:** 1 Centre for Innate Immunity and Infectious Diseases, Hudson Institute of Medical Research, Clayton, VIC 3168, Australia; gabriel.laghlali@hudson.org.au (G.L.); kate.lawlor@hudson.org.au (K.E.L.); 2 Department of Molecular and Translational Sciences, Monash University, Clayton, VIC 3168, Australia; 3 Master de Biologie, École Normale Supérieure de Lyon, Université Claude Bernard Lyon I, Université de Lyon, 69007 Lyon, France

**Keywords:** Influenza A virus, cell death, inflammation, pathogenesis

## Abstract

Influenza A virus (IAV) is a major concern to human health due to the ongoing global threat of a pandemic. Inflammatory and cell death signalling pathways play important roles in host defence against IAV infection. However, severe IAV infections in humans are characterised by excessive inflammation and tissue damage, often leading to fatal disease. While the molecular mechanisms involved in the induction of inflammation during IAV infection have been well studied, the pathways involved in IAV-induced cell death and their impact on immunopathology have not been fully elucidated. There is increasing evidence of significant crosstalk between cell death and inflammatory pathways and a greater understanding of their role in host defence and disease may facilitate the design of new treatments for IAV infection.

## 1. Introduction

Influenza A virus (IAV) infects approximately 3 to 5 million people each year, and the World Health Organisation estimates that 290–650 thousand deaths occur annually worldwide, with the young, old, immunocompromised and individuals with underlying lung and heart conditions at the highest risk. One of the most significant pandemics was the H1N1 Spanish Influenza, with deaths estimated at 50–100 million people between 1918 and 1920 [1]. IAV possesses a wide diversity of natural reservoirs, including aquatic birds and swine. Transmission of IAV from birds to human occurs sporadically, however, in most cases, the infection cannot disseminate from human to human. For example, avian H7N9 IAV emerged in humans in 2013 and there have now been over 1500 laboratory-confirmed cases, with a mortality rate of approximately 40%. However, experts predict that a pandemic involving H7N9 is imminent [2].

Current treatments to protect against IAV involve yearly vaccination and antiviral therapies. IAV vaccines target specific strains of IAV that are predicted to predominate in the coming season. Vaccines are likely to protect poorly against an emerging strain of IAV, such as in the event of a pandemic. Antivirals, such as oseltamivir, target the virus and show limited efficacy due to IAV resistance and the need to administer within the first 48 h of infection [3]. A recent Cochrane review, analysing large sets of unpublished industry studies, concluded that anti-viral drugs have no effect on mortality, limited impact on hospital stay duration and correlated with a number of side effects including nausea and vomiting [4]. There is an urgent need to develop new treatments for severe IAV infections that are broadly active against a range of IAV strains and potentially target the host to protect against the development of fatal disease.

The severity of IAV infection can vary amongst individuals. The infection can be asymptomatic, lead to moderate symptoms, such as fever and cough, or in the case of severe infection, it can induce respiratory distress syndrome (ARDS)-like disease, leading to respiratory and multiorgan failure, and even death [5]. IAV infects respiratory epithelial cells of the upper-respiratory tract, where it is generally confined in cases of mild infections involving seasonal strains. In the case of severe IAV infections, the virus can reach the lower respiratory tract, in which it infects pulmonary epithelial cells [6]. Infection of epithelial cells and alveolar macrophages results in the induction of inflammatory and cell death pathways that play important roles in mediating resolution of the infection. However, severe IAV infections are associated with excessive inflammation and damage to the epithelium, which leads to pulmonary edema and vascular leakage, hallmark features of ARDS [5]. In addition, secondary bacterial infections can greatly increase mortality. This review will discuss the induction and interplay between inflammation and cell death during IAV infection.

## 2. Induction of Inflammation during IAV Infection

Airway epithelial cells and resident alveolar macrophages are the primary targets of IAV infection. While infection of epithelial cells leads to viral propagation, alveolar macrophages (with the exception of some avian H5) provide a ‘dead end’ whereby infectious virus is not released [7,8,9]. Importantly, epithelial cell and macrophage infection results in the induction of inflammatory and antiviral pathways [7,10]. Leukocytes are recruited into the lungs, such as neutrophils, inflammatory macrophages, dendritic cells (DCs) and natural killer (NK) cells during the early phase of infection, followed by T and B lymphocytes 5–7 days post-infection [11,12,13].

**Figure 1:**
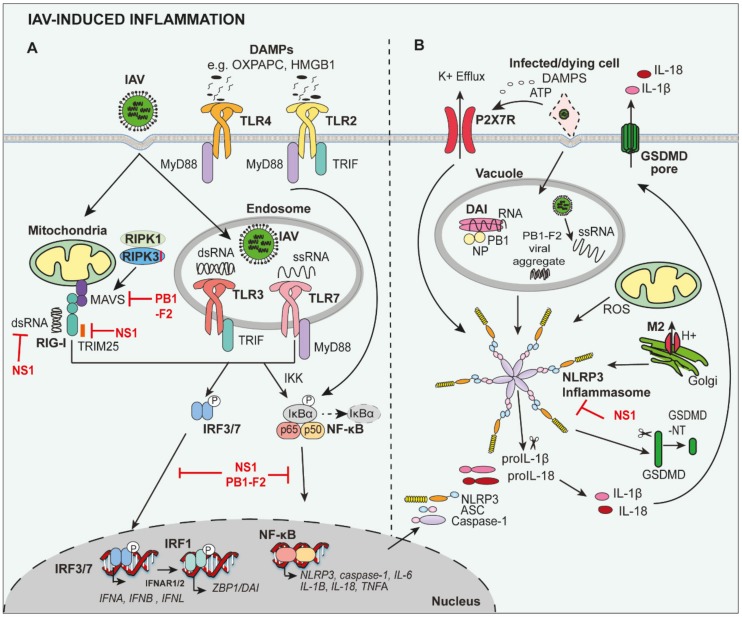
Anti-viral and inflammatory pathways during IAV infection. (**A**) Induction of inflammation involves the activation of patter recognition receptors, including toll-like receptors (TLR) and retinoic acid-inducible gene I (RIG-I) pathways. Recognition of viral RNA by TLR3/7 and RIG-I, as well as damage-associated molecular patterns (DAMPs), such as high Mobility Group Box 1 (HMGB1), by TLR2/4 leads to the activation of the transcription factors nuclear factor kappa-light-chain-enhancer of activated B cells (NF-κB) and interferon regulatory factor (IRF)3/7. Inflammation and anti-viral responses are mediated by cytokines (e.g., IL-6, TNFα, pro-IL-1β and pro-IL-18), chemokines and anti-viral type I and III interferons (IFNs), respectively. The IAV proteins NS1 and PB1-F2 can inhibit RIG-I, as well as IRF3/7- and NF-κB-mediated responses. (**B**) NF-κB-mediates upregulation of NLRP3 inflammasome components, namely, NLRP3, caspase 1, pro-IL-1β and pro-IL-18, to provide the first “priming” signal for NLRP3 activation. A second signal results in the assembly of the NLRP3 inflammasome complex, allowing the processing and cleavage of pro-IL-1β and pro-IL-18 precursors into their bioactive forms, IL-1β and IL-18, by caspase-1. Gasdermin D (GSDMD) is also cleaved by caspase-1 and the N-terminal domain of GSDMD (GSDMD-NT) inserts into the plasma membrane and forms a pore to facilitate the release of bioactive IL-1β and IL-18. Signal 2 is provided by imbalances in ionic concentrations in intracellular vesicles as a result of PB1-F2-mediated production of mitochondrial reactive oxygen species (ROS), M2 ion channel formation and H+ efflux in the golgi apparatus, activation of the P2X7 purinergic receptor following release of extracellular adenosine 5′-triphosphate (ATP) from infected/dying cells, as well as DAI/ZBP1 sensing of viral RNA. The IAV NS1 protein has been shown to inhibit NLRP3 inflammasome responses.

IAV pathogen-associated molecular patterns (PAMPs) are detected by pattern recognition receptors (PRRs), including retinoic acid-inducible gene-I-like receptors (RLRs) and toll-like receptors (TLR) [14] (Figure 1A). TLRs, such as TLR3 and TLR7, and the RLR retinoic acid-inducible gene I (RIG-I), recognise viral single-stranded RNA (ssRNA) or the ‘panhandle’ structure of the (sub)genomic RNA/double-stranded RNA (dsRNA) in the endosomal compartment and cytosol, respectively (Figure 1A). The activation of nuclear factor kappa-light-chain-enhancer of activated B cells (NF-κB) leads to the production of pro-inflammatory cytokines and chemokines, such as interleukin (IL)-6, tumour necrosis factor (TNF)α, monocyte chemoattractant protein-1 (MCP-1; C-C Motif Chemokine Ligand 2 (CCL2)), pro-IL-1β and pro-IL-18, whereas interferon regulatory factor (IRF)3 and IRF7 result in the production of anti-viral type I and III interferons (IFNs) [15].

The cytosolic PRR, NOD-, LRR- and pyrin domain-containing protein 3 (NLRP3) is additionally activated during IAV infection, leading to the formation of a multiprotein inflammasome complex which enzymatically matures inactive precursors, pro-IL-1β and pro-IL-18, into bioactive IL-1β (p17) and IL-18 (p18), and leads to a lytic form of cell death, termed pyroptosis [16,17]. NLRP3 activation is tightly regulated by the requirement for two signals. Firstly, expression of pro-IL-1β, pro-IL-18, caspase-1 and NLRP3 are upregulated by NF-κB following detection of viral RNA by RIG-I, TLR3 and/or TLR7 (Figure 1A). A second signal then triggers the assembly and activation of the NLRP3 inflammasome complex (comprises NLRP3, ASC and pro-caspase-1), and induces proximity-mediated dimerization and activation of caspase-1 to process pro-IL-1β and pro-IL-18 (Figure 1B), as well as cleave Gasdermin D (GSDMD) to induce pyroptotic cell death (discussed in detail below). For seasonal and pathogenic IAV, the second activation signal is triggered by viral RNA and the IAV matrix protein 2 (M2) forms a pore in the Golgi apparatus, altering the intracellular concentration of hydrogen ions (H^+^). In addition, extracellular adenosine 5′-triphosphate (ATP) released from dead/dying cells activates the P2X purinoceptor 7 (P2X7) receptor, resulting in potassium efflux, as well as reactive oxygen species (ROS) generation, which can activate NLRP3 inflammasome assembly [18,19,20]. Importantly, the IAV PB1-F2 protein from pathogenic H1N1 and H7N9, but not seasonal IAV, can induce mitochondrial ROS production, which also contributes to NLRP3 activation [21,22]. More recently, Z-DNA binding protein-1 (ZBP1), also known as DNA-dependent activator of IFN-regulatory factors (DAI), has been shown to sense viral RNA to trigger NLRP3 inflammasome assembly [23,24].

Despite the fact that IAV is sensed by PRRs to promote anti-viral responses, the IAV non-structural (NS)1 protein has been shown to limit host inflammatory responses to protect the replicative niche (Figure 1). For example, RIG-I activation is impaired by NS1 sequestering dsRNA or via its interaction with RIG-I regulators Riplet or TRIM25 [25,26]. In addition, it has been shown that NS1 can interact directly with the NLRP3 inflammasome to suppress responses in macrophages [27,28,29].

## 3. Inflammatory Responses in Mediating Host Defence and Promoting Disease during Severe IAV Infection

### 3.1. PRRs Mediate Protective and Detrimental Inflammation

As discussed above, TLR3, TLR7 and RIG-I recognise viral RNA, leading to the activation of NF-κB- and IRF3/7-mediated inflammatory and anti-viral responses. While IAV clearance is delayed in mice lacking RIG-I, or the downstream adaptor mitochondrial antiviral-signalling protein (MAVS), no significant differences in the overall survival of mice was observed following lethal A/Puerto Rico/8/34 (PR8) H1N1 infection [30,31,32]. TLR7 deficiency also did not alter viral loads or susceptibility, despite impairments in NK cell responses [32,33]. In contrast, TLR3-deficient mice have been shown to be more susceptible to highly pathogenic avian influenza (HPAI) H5N1 infection [34], but are surprisingly protected from A/Scotland/20/74 (H3N2) and 2009 pandemic H1N1 infection [34,35], suggesting that the contribution of TLR3 responses to IAV infection may vary amongst strains. Interestingly, TLR2 and TLR4, which both signal via the adaptor MyD88, have been shown to be engaged by oxidized 1-palmitoyl-2-arachidonyl-sn-glycero-3-phospholcholine (OxPAPC) and high mobility group box 1 (HMGB1) released from dying cells (Figure 1A). Mice lacking TLR4 were shown to be resistant to PR8 infection, whereas TLR2-deficent mice were susceptible akin to wild-type animals [36,37]. However, therapeutic targeting of either TLR2 or TLR4 from day two post-infection provided significant protection, suggesting that both TLR2 and TLR4 contribute to disease pathogenesis driven by damage-associated molecular patterns (DAMPs) [37]. Mice with impairments in multiple PRR pathways, such as *Trif*^–/–^ (TIR-domain-containing adapter-inducing interferon-β; selective TLR3 and TLR4 adaptor; [38]), *Myd88*^–/–^ *Trif*^–/–^ (all TLR signalling affected; [38]), *Myd88*^–/–^ *Mavs*^–/–^ [39], *Tlr7*^–/–^ *Mavs*^–/–^ [32] or *Tlr3*^–/–^ *Tlr7*^–/–^ [38], are more susceptible to PR8 infection with reduced anti-viral responses and increased viral loads. Similarly, mice lacking MyD88 are also more susceptible to PR8, H5N1 and pH1N1 infection [34,38]. Therefore, the RIG-I and TLR pathways compensate for the loss of another, for example TLR7, suggesting that redundancy exists between the PRR pathways.

Mice lacking components of the NLRP3 inflammasome complex—NLRP3, ASC and caspase-1, initially demonstrated the critical role of the inflammasome in eliciting protective immunity during PR8 infection [18,20,40]. Therapeutic targeting of NLRP3 from day 1 with the potent NLRP3 inhibitor MCC950 also confirmed the requirement of inflammation to combat early disease, as mice were rendered more susceptible to HKx31 (H3N2) and PR8 infection by MCC950 treatment. However, MCC950 treatment from day 3 or day 5 post-infection respectively, unexpectedly limited hyperinflammation and prolonged survival [13]. These results suggest a biphasic role for NLRP3 inflammasomes in severe IAV infection. Interestingly, the drugs probenecid and AZ11645373, which target the P2X7 receptor upstream of NLRP3 activation (Figure 1B) in a less potent manner, also provided protection against HKx31 and PR8 infection when treatment was commenced prior to or following the development of severe disease [41], and thus potentially reduced the risk of adverse effects from potent NLRP3 inhibitor drugs. Collectively, TLR and NLRs may play a balancing act of mediating protective versus detrimental inflammation during severe IAV disease. Downstream recognition of virally released DAMPs by TLR2, TLR4 and NLRP3 may also potentially amplify inflammatory responses.

### 3.2. Pro-Inflammatory Cytokines and the ‘Cytokine Storm’

The production of high concentrations of a range of cytokines, termed a ‘cytokine storm’, induces damage to the lung epithelium, as well as multiple organ failure, resulting in enhanced morbidity and mortality. Elevated levels of IL-1β, IL-18, TNFα and IL-6 are prognostic markers of poor outcomes in patients infected with H7N9 IAV [42,43]. Mice lacking IL-6 [44,45], IL-1 receptor (IL-1R; binds IL-1β and IL-1α) [40,46], IL-18 receptor (IL-18R) [47,48] and the type I IFN receptor IFNAR1 [49] are more susceptible to PR8 infection. In contrast, mice lacking the MCP-1 receptor C-C chemokine receptor type 2 (CCR2) are protected from PR8 infection with reduced inflammatory macrophage infiltration, epithelial cell apoptosis and lung injury [50,51,52,53]. While type I IFN signalling has been shown to be critical for antiviral responses following infection with two human H5N1 isolates [54], a lack of IL-1R, IL-6, macrophage inflammatory proteins-1α (MIP-1α/CCL3) or TNF receptor 1 (TNFR1) had limited or no impact [55], suggesting that individual cytokines or pathways do not mediate severe H5N1 disease. As discussed above, IL-1β and IL-18 are potent NLRP3 inflammasome-activated pro-inflammatory cytokines that bind the cell surface receptors IL-1R and IL-18R respectively, and induce further amplification of NF-κB-dependent cytokine production, e.g., IL-6, TNFα, IL-8, pro-IL-1β and pro-IL-18. However, despite our emerging understanding of NLRP3 inflammasome activity in IAV, currently, the role of IL-1β during IAV infection has not been directly examined. In addition, the contribution of IL-18 to disease is unclear, as mice lacking the IL-18 receptor (IL-18R) have been reported to be more [47,48] or less [56] susceptible to PR8 infection. In addition, it is possible that particular cytokines examined largely by using genetic models may play an early role in host defence, but later dysregulation could promote the development of fatal disease.

### 3.3. Cellular Immune Response

Severe IAV infections in humans and mice are characterised by the infiltration of large numbers of leukocytes, such as macrophages and neutrophils, into the airways. Studies have demonstrated the importance of both neutrophils and macrophages in limiting viral replication during mild BJx109 (H3N2), moderate HKx31 (H3N2) and severe PR8 (H1N1) infection [8,11,57,58]. Natural killer (NK) cells can induce apoptosis of infected cells to limit viral replication [59,60]. The production of MCP-1 by epithelial cells results in the significant infiltration of Ly6C+ inflammatory macrophages several days following IAV infection [13,49], potentially prolonging and/or amplifying the inflammatory response to drive disease development [50,51,52]. In addition, innate immune cells, such as DCs and neutrophils, are important for the development of adaptive immune responses, which can ultimately play critical roles in resolving the infection [11].

## 4. Cell Death Pathways and Their Activation during IAV Infection

The many and varied cell death pathways—pyroptosis, extrinsic apoptosis and necroptosis, intrinsic apoptosis and secondary necrosis—are distinct but highly complex in nature (see Table 1). These pathways have been implicated in IAV responses in key pathogenic cells, including epithelial cells and macrophages (Table 2). However, a number of caveats in the design of these studies suggests that a thorough re-evaluation of the contribution of cell death to IAV infection is warranted. For example, studies have been largely performed using the PR8 H1N1 strain, which was mouse-adapted by >300 sequential passages in mouse lung [61], which most likely resulted in the acquisition of mutations associated with increased replication efficiency in the respiratory tract and/or evasion of innate host responses. Indeed, PR8 is highly virulent for mice and poorly infects macrophages, in comparison to HKx31 (H3N2) of intermediate virulence, as well as human seasonal H3N2 and H1N1 IAV [7,8,62]. A number of studies have also examined cell death pathways in murine embryonic fibroblasts (MEFs) that are somewhat resistant to IAV replication [23,63,64]. Fibroblasts have been shown to not be physiologically relevant upon IAV infection in vivo, compared to epithelial cells and alveolar macrophages [7,65,66]. A final potential issue is that the human epithelial cancer cell lines (e.g., adenocarcinoma A549 type II alveolar epithelial cells) used to examine IAV-mediated cell death, have been shown to be more resistant to IAV cell death in comparison to primary cell counterparts [33], potentially due to a lack of receptor-interacting serine/threonine-protein kinase 3 (RIPK3) expression that can trigger extrinsic apoptotic and necroptotic cell death signalling, and coordinate NLRP3 inflammasome activation [67,68,69,70,71,72,73]. Studies in primary airway epithelial cells and macrophages with a range of IAV strains will be particularly important to fully elucidate the role of cell death pathways during IAV.

## 5. NLRP3 Inflammasome-Associated Pyroptosis in IAV Infection

**Figure 2:**
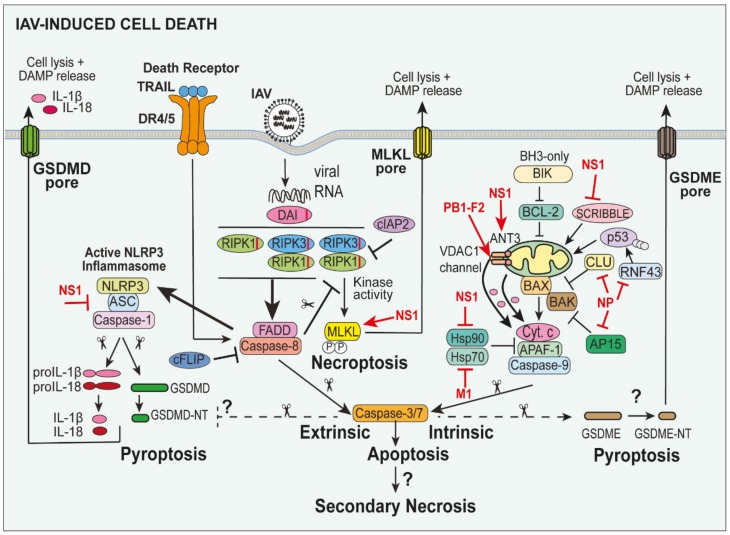
Cell death pathways during IAV infection. Extrinsic apoptosis is triggered when death receptors (e.g., death receptor (DR4)/5) are engaged by their ligands (e.g., TNF-related apoptosis-inducing ligand (TRAIL)), leading to the activation of a Fas associated via death domain (FADD)-caspase-8 complex. Z-DNA-binding protein 1 (ZBP1; also known as DAI) can also sense viral RNA and interact via RHIM–RHIM interactions with RIPK1/3 to also form a FADD-caspase-8 death-inducing complex. Autoactivation of the initiator caspase, caspase-8, subsequently induces the cleavage and the activation of effector caspases, caspase-3/7, triggering cellular demise. Induction of necroptosis, when caspase-8 levels are low or chemically inhibited, is also mediated by the kinases, receptor-interacting protein kinase (RIPK)1 and RIPK3. RIPK3 critically phosphorylates and activates mixed lineage kinase domain like pseudokinase (MLKL), causing it to oligomerize and insert into the plasma membrane, forming a pore that allows the release of damage-associated molecular patters (DAMPs) and cellular breakdown. In the case of extrinsic apoptosis, the cellular cellular inhibitor of apoptosis (cIAPs)1/2) and caspase-8 inhibitor c-FLIP ((FADD-like IL-1β-converting enzyme)-inhibitory protein) can inhibit apoptosis induction, while necroptosis can reportedly be inhibited by cIAP1/2 or via the caspase-8-mediated cleavage of RIPK1. Of note, the IAV protein NS1 can enhance MLKL oligomerization and membrane translocation. Intrinsic mitochondrial apoptosis is triggered by cellular stressors, including the IAV proteins NS1, PB1-F2, NP and M1, that activates BAX/BAK pore and voltage-dependent anion-selective channel 1 (VDAC1) channel formation to impair mitochondrial outer membrane integrity and allow the release of cytochrome *c* (Cyt. *c*) into the cytosol. Cyt. *c* release triggers assembly of the Cyt. *c*/apoptotic protease activating factor 1 (APAF-1)/caspase-9 apoptosome complex and active caspase-9 cleaves caspase-3/7 to induce cell death. In the case of NLRP3 inflammasome activation in response to viral pathogen-associated molecular patterns (PAMPs; see Figure 1), active caspase-1 cleaves gasdermin D (GSDMD; mouse Asp 276) to release the N-terminal domain of GSDMD (GSDMD-NT), which oligomerizes within the membrane and forms a pore to facilitate the release of IL-1β and IL-18, as wells as the release of DAMPs, such as high-mobility group box 1 (HMGB1). Interestingly, with new knowledge on novel activities for apoptotic caspase-3, it remains to be seen in IAV infection if caspase-3 cleaves GSDMD (Asp 88) to inhibit its pore-forming potential, and/or whether during apoptosis caspase-3 can cleave gasdermin E (GSDME) to release the pore-forming N-terminal domain of GMDME (GSDME-NT) and induce pyroptosis.

Pyroptotic cell death is a well-characterised inflammatory form of cell death induced by NLRP3 inflammasome activation upon viral infection (Figure 1 and Figure 2). The active NLRP3 inflammasome complex induces caspase-1 dimerisation and autoactivation and subsequent cleavage of GSDMD between the autoinhibitory C-terminal and active N-terminal domains at Asp 276 in mice and Asp 275 in humans [87]. The active N-terminal GSDMD p30 subunits (GSDMD-NT) induces the lytic release of cytoplasmic contents and DAMPs (e.g., IL-1α and HMGB1) by inserting into the lipid membrane and oligomerizing to form transmembrane pores (~10–20 nm) [88]. The GSDMD pore also facilitates the release of pro-inflammatory IL-1β and IL-18 from the cell to potently promote inflammation [89,90].

Of note, GSDMD can be cleaved independently of caspase-1 under certain conditions, suggesting that the degree of caspase-1 activation may not directly correlate with pyroptosis. Neutrophil elastase has also been shown to cleave neutrophil GSDMD [91,92]. During Gram-negative bacterial infections, human caspase-4/5 or murine caspase-11 recognise lipopolysaccharide (LPS) and potently cleave and activate GSDMD-mediated lysis [87,93]. More recently, the YopJ effector of Gram-negative *Yersinia* has been shown to inhibit transforming growth factor beta-activated kinase 1 (TAK1) and IKK to induce caspase-8-mediated GSDMD cleavage, independent of caspase-1 [85,94,95]. In contrast, the apoptotic effector caspase-3 can cleave and inactivate GSDMD-NT (at Asp 87) to limit pyroptosis [96,97]. Crosstalk between inflammatory and apoptotic cell death signalling is also further highlighted by the fact that caspase-1 has also been shown to induce caspase-3-mediated apoptosis and secondary necrosis in GSDMD-deficient cells [98,99].

As discussed above, NLRP3 inflammasome activity plays a biphasic role in IAV infection, which is protective early and damaging during the later stages of severe disease [13]. However, the cell-by-cell contribution of inflammasome activation and cell death during IAV infection is less resolved. A number of groups have shown that PR8 infection of murine bone-marrow-derived macrophages (BMDMs) induces caspase-1 cleavage [24,85,86]; however, GSDMD cleavage has not been reported. While *Ripk3^−/−^* BMDMs (defective in extrinsic cell death; see below) were resistant to PR8-induced cell death, lack of NLRP3, caspase-1 or GSDMD did not significantly impact cell death [85]. These results could suggest that there is a bifurcation of the inflammasome signalling pathway, and that apoptosis is the dominant mode of cell death; although, the results could also be attributed to the poor ability of the PR8 strain to infect and induce cell death in murine macrophages in vitro [7,8,62]. Interestingly, while PR8 infection fails to induce GSDMD-mediated pyroptosis, the PB1-F2 protein from PR8, HKx31 and H7N9 IAV can activate NLRP3-caspase-1-mediated cleavage, IL-1β activation and pyroptotic-like cell death in BMDMs [21,22]. In human studies, monocyte-derived macrophages were resistant to cell death following infection with wild-type PR8. However, mutants lacking NS1 were able to induce greater cell death, as well as IL-1β and IL-18 release, which were reduced by treatment with a caspase-1 inhibitor [84]. Importantly, GSDMD cleavage has been reported in human precancerous respiratory epithelial cells (PL16T cells) following PR8 infection [33] (Table 2). Interestingly, in this study, treatment of human primary bronchial epithelial cells and PL16T cells with caspase-3 or caspase-1 inhibitors suggested that apoptosis was dominantly induced in the early phases of IAV infection but shifted to pyroptosis at later phases of infection. Currently, the specific role of GSDMD-mediated pyroptosis in modulating IAV replication and disease outcomes has not been directly studied.

## 6. Apoptosis in IAV Infection

Apoptosis is a highly regulated form of programmed cell death that promotes the removal of damaged, superfluous and infected cells in an immunologically silent manner. Apoptotic cell death is characterised by cell shrinkage, DNA fragmentation, membrane blebbing, chromosomal condensation and caspase-3 and caspase-7 activation (Table 1). Apoptosis/DNA fragmentation was first identified in the early 1990s in Madin-Darby Canine Kidney (MDCK) cells [100] and later observed in human and murine primary bronchial epithelial cells following PR8 infection [33,101]. Apoptosis was also noted in human monocytes [102] and subsequently in macrophages that do not typically support IAV replication [84,103,104]. In addition to these key cells, human monocyte-derived DCs [105], plasmacytoid DCs [106], NK cells [107,108], as well as human and murine neutrophils [109,110], have also been shown to undergo apoptosis following IAV infection; however, the pathways involved have not been well studied in these cells. It is clear that apoptosis is widely triggered as a host defence mechanism to limit viral replication and the time for the production of viral proteins and virion formation/release. The roles of extrinsic “death receptor-mediated” and intrinsic “mitochondrial and BCL-2 family-regulated” forms of apoptosis in IAV infection are discussed below.

### 6.1. Extrinsic Apoptosis in IAV

The extrinsic pathway of apoptosis is initiated by the binding of death ligands of the TNF receptor superfamily, such as TNFα, Fas ligand (FasL) and TNF-related apoptosis-inducing ligand (TRAIL) to their receptors, TNFR1, Fas, death receptor (DR)4/DR5 respectively, to mediate Fas-associated protein with death domain (FADD)/caspase-8-dependent apoptosis [111,112] (Figure 2). In particular, TNFα, which is released by IAV-infected cells, binds to TNFR1 to induce the formation of a TNFR1 signalling complex (complex 1) comprising TNFR1-associated death domain protein (TRADD), RIPK1, the adaptor TNF receptor-associated factor 2 (TRAF2) that allows the recruitment of the E3 ubiquitin ligases cellular inhibitor of apoptosis (cIAP) proteins, cIAP1 (Birc2) and cIAP2 (Birc3), and the linear ubiquitin chain assembly complex (LUBAC; comprises HOIL, HOIP and SHARPIN) [113,114,115]. The cIAPs and LUBAC ubiquitylate RIPK1 to prevent its association with FADD and caspase-8 and promote a downstream signalling cascade, culminating in NF-κB-mediated activation of pro-survival signals (e.g., cellular FLICE-like inhibitory protein (c-FLIP) upregulation) and cytokine/chemokine production [114]. When pro-survival responses are compromised (e.g., loss of the IAPs), TRADD and RIPK1/RIPK3 can associate with FADD and pro-caspase-8 to form a secondary complex, which can drive apoptosis via autoactivation of caspase-8 and the resultant activation of downstream caspase-3 and -7 (Figure 2) [116].

In the context of IAV, extrinsic apoptosis and caspase-8 cleavage has been observed in human primary type I-like alveolar epithelial cells following H5N1 and seasonal H1N1 [74], as well as in human monocytes (H7N9) [83], monocyte-derived macrophages [84] and murine BMDMs [24,85,86] following PR8 infection.

Death receptor-mediated apoptosis has been shown to potentially augment epithelial cell death during IAV infection. FasL upregulation, FADD phosphorylation and downregulation of the inhibitor c-FLIP has been reported in A549 cells following the 2009 pandemic H1N1 infection [117]. Furthermore, treatment of PR8-infected mice with a recombinant decoy receptor for FasL (Fas-Fc) has been shown to improve survival [118]. TRAIL is also reportedly expressed by PR8-infected alveolar macrophages to induce epithelial cell apoptosis [53]. Indeed, treatment from day three post-infection with an anti-TRAIL antibody improved the survival of PR8-infected mice and reduced alveolar epithelial cell apoptosis and alveolar leakage, despite enhancing viral loads. In line with this study, alveolar macrophages were shown to upregulate TRAIL and alveolar epithelial cells the death receptor DR5, in an IFNβ/IFNAR1-dependent manner following PR8 infection [119]. In this study, TRAIL was also shown to be upregulated in alveolar macrophages from patients with 2009 pandemic H1N1-induced ARDS. Notably, TRAIL expression on NK cells and CD4^+^ and CD8^+^ T lymphocytes has also been observed in the lungs of PR8-infected mice [120], suggesting that lymphocytes have the potential to facilitate TRAIL-mediated cell death during IAV infection. These studies highlight a role for macrophages (and perhaps NK and T lymphocytes) in prompting apoptosis in by-stander cells, such as epithelial cells.

### 6.2. Intrinsic “Mitochondrial-Dependent” Apoptosis in IAV Infection

Intrinsic “mitochondrial-dependent and BCL-2 family protein-regulated” apoptosis ensues upon exposure of the cells to stressors, such as growth factor withdrawal, ROS, DNA damage and metabolic changes. It is irreversibly activated when BCL-2-associated X protein (BAX) and/or BCL-2 homologous antagonist/killer (BAK) insert into the mitochondrial outer membrane causing permeabilisation [121] (Figure 2). BAX/BAK activation is tightly regulated by the balance of pro-survival (e.g., B-cell lymphoma (BCL)-2, BCL-XL, BCL-W, induced myeloid leukemia cell differentiation protein (MCL-1)) and pro-apoptotic BH3-only proteins (e.g., BCL-2-interacting killer (BIK), B-cell lymphoma 2 interacting mediator of cell death (BIM), p53 upregulated modulator of apoptosis (PUMA), NOXA, BH3-interacting death domain agonist (BID)) [122]. Upon BAX/BAK-induced mitochondrial outer membrane permeabilisation (MOMP), cytochrome *c* (cyt. *c*) is released into the cytosol, and along with apoptotic protease activating factor 1 (APAF-1) and caspase-9, forms the “apoptosome”, which, in turn, activates caspase-9, resulting in cleavage and activation of downstream caspase-3 and caspase-7 [123,124,125]. It is worth mentioning that there is the potential for crosstalk between intrinsic and extrinsic cell death, whereupon caspase-8 can cleave and truncate BID to activate mitochondrial apoptosis, and MOMP facilitates the release of second mitochondrial activator of caspase (SMAC), the natural antagonist of the cIAP proteins, and related family member X-linked IAP (XIAP) [126,127].

Intrinsic BAX/BAK-dependent apoptosis or caspase-9 cleavage has been reported following H5N1 and seasonal H1N1 infection of human primary type I-like alveolar epithelial cells [74], as well as PR8 infection of A549 cells [75,76], primary murine tracheal cells [77] and H7N9-infection of human blood-derived monocytes [83] (Table 2). Mechanistically, the 2009 pandemic H1N1 infection of A549 cells has been shown to promote apoptosis by downregulation of pro-survival BCL-XL [117]. Over-expression of pro-survival BCL-2 in MDCK cells limits apoptosis and A/WSN/33 (WSN/33) replication, as does a lack of the essential apoptotic effector BAX [128]. Conversely, BAK deficiency in MDCK cells increased apoptotic cell death and WSN/33 production and infection naturally depleted BAK to preserve the replicative niche [128]. Of note, WSN/33 is unusual as it is neutrotropic and can undergo HA cleavage activation in vitro without the addition of exogenous trypsin. In line with a pro-apoptotic role for BAX, expression of the BAX-selective pro-apoptotic BH3-only protein BIK is induced following PR8 infection to promote apoptosis and viral replication in murine tracheal epithelial cells [77]. To be more specific, BIK promoted the cytoplasmic export of viral ribonucleoprotein and cleavage of NP and M2 IAV proteins. Consequently, mice lacking BIK were protected from the lethal PR8 infection with improved survival associated with reduced inflammation and viral loads in the lung. Collectively, these studies suggest that BAX/BAK-dependent apoptosis is activated, particularly in epithelial cells following IAV infection.

The IAV proteins PB1-F2, NS1, NP and matrix 1 (M1) have been shown to regulate intrinsic apoptosis via a number of unique mechanisms (Figure 2). PB1-F2, for example, has been shown to interact with mitochondrial voltage-dependent anion channel 1 (VDAC1) and adenine nucleotide translocator 3 (ANT3) proteins to induce VDAC1 channel formation in the mitochondrial membrane and allow the efflux of cyt. *c* [129]. The IAV NS1 protein has also been shown to induce cyt. *c* release from mitochondria and activate caspase-9 [130,131]. Similarly, NS1 proteins from H5N1 and H3N2 viruses have been shown to interact with the protein chaperone Heat shock protein (Hsp)90 and inhibit its interaction with APAF-1 to allow optimal formation of the apoptosome in A549 cells [132]. Consistent with these results, the PR8 virus lacking NS1 induced less caspase-3 activation in human epithelial cells [33]. On the contrary, NS1 can bind to the pro-apoptotic protein Scribble to limit cell death and it is proposed that this may prevent the early induction of apoptosis [133]. The IAV NP protein also exerts pro-apoptotic activities in epithelial cells by interacting with anti-apoptotic proteins, Clusterin (CLU) [134] and apoptosis inhibitor protein 15 (AP15) [135], to reduce their association with BAX (and BAK), as well as binding the RING-type E3 ubiquitin ligase RNF43 that typically destabilises pro-apoptotic p53 [136]. Lastly, the IAV M1 protein can bind to Hsp70, which like Hsp90, inhibits APAF-1 and promotes apoptosome-mediated caspase-9 activity [137]. Hence, although IAV proteins may potentially limit early induction of apoptosis, overall, IAV activates intrinsic apoptosis via a number of mechanisms. Intriguingly, since apoptosis promotes reduced virus production, there has been growing interest in harnessing selective BH3-mimetics targeting pro-survival members to trigger the premature death of infected cells [138].

### 6.3. Necroptosis Pathways in IAV

As discussed above, IAV infection can lead to extrinsic caspase-8-dependent apoptosis. However, if caspase-8 activity (and cell death repression) is compromised, death receptor (e.g., TNFR1) and PRRs (e.g., TLRs) ligation can signal a caspase-independent form of cell death, termed necroptosis [139,140] (Figure 2). Necroptosis, unlike apoptosis, is regulated by the kinase activities of RIPK1 and RIPK3, where the kinase RIPK3 critically phosphorylates the pseudokinase Mixed Lineage Kinase domain-Like (MLKL) and induces a conformational change, exposing the N-terminal four-helix bundle (4HB). Subsequently, MLKL oligomerises and inserts into the plasma membrane to destroy the integrity of the membrane and allow the release of DAMPs [141].

Recent studies have detected necroptosis effector MLKL or its phosphorylated active form (pMLKL) following PR8 infection in murine primary type I alveolar epithelial cells [82], lung fibroblasts [86], BMDMs [82,86], as well as in DCs following seasonal H1N1 and the 2009 pandemic H1N1 [142] (Table 2). Likewise, necroptotic signalling responses were observed in human blood-derived monocytes upon H7N9 infection and were sufficient to promote pro-inflammatory cytokines gene expression, e.g., IFNβ, IL-6, and RANTES/CCL5 and costimulatory protein CD80, CD83 and CD86 expression on monocyte-derived DC and T cell proliferation [83]. Patients with fatal ARDS-like H7N9 disease also display increased total levels of necrosome components RIPK1, RIPK3 and MLKL in lung tissue samples, as well as active phosphorylated forms of RIPK3 and MLKL [143]. Similarly, pMLKL has also been detected in lung tissues following PR8 infection of mice [82]. Consistent with these findings, the IAV NS1 protein has been reported to induce necroptosis by interacting with MLKL and increasing oligomerisation and membrane translocation in HEK293T cells [69]. Contradictorily, genetic deletion of MLKL in BMDMs does not alter cell death following PR8 infection [85]. However, these results have not been confirmed with other IAV strains, in particular those capable of infecting macrophages more efficiently.

A recent study by Zhang et al. [144] identified that Z-RNA from replicating IAV activates DAI/ZBP1 in the nucleus of infected MEFs, triggering RIPK3-mediated MLKL activation. MLKL induced nuclear envelope rupture, subsequent leakage of DNA into the cytosol and necroptosis [144]. While the authors detected Z-RNA in IAV-infected A549 cells and in the LET1 murine lung alveolar type I line, the role of nuclear MLKL activation upon IAV-induced ZBP1 signalling in these physiologically relevant cell types requires further investigation.

### 6.4. DAI-RIPK3-Driven Apoptotic Caspase-8 and Necroptotic MLKL during IAV Infection

Examination of extrinsic cell death pathways in IAV has revealed evidence that both extrinsic apoptosis and necroptosis pathways can be activated in parallel. IAV infection has been observed to induce RIPK3 signalling and caspase-8-dependent apoptosis, as well as RIPK3 kinase-dependent MLKL-mediated necroptosis in MEFs (Figure 2) [144,145].

Studies have revealed that while *Ripk3*^−/−^ and *Mlkl*^−/−^ *Fadd*^−/−^ mice are hypersusceptible to PR8 infection and display increased viral loads [145,146], following sublethal PR8 infection, *Mlkl*^−/−^ mice exhibit no gross differences in susceptibility [144,145]. However, it has recently been shown that mice lacking MLKL display improved survival following lethal PR8 infection [144]. Notably, protection from lethal PR8 in *Mlkl*^−/−^ was not associated with changes in viral loads but rather, reduced neutrophil numbers and neutrophil extracellular traps (NETs). The results therefore point to an important protective role of FADD-caspase-8-mediated apoptotic signalling during IAV infection and MLKL-mediated necroptotic signalling inducing damaging inflammation. However, another study showed that RIPK3-deficient mice were protected from H7N9 infection, and displayed reduced levels of cytokines in the airways, such as IL-1β and IL-6, but exhibited no reduction in pMLKL levels [147], suggesting that the role of RIPK3 in promoting protection or disease may be strain-specific. Of note, RIPK3 has reportedly been suggested to interact with MAVS to promote type I IFN responses during IAV infection, suggesting that it can also play a role in inflammatory/host defence in a manner separable from cell death [146] (Figure 1A).

How IAV signals to RIPK3 to trigger apoptotic and necroptotic signalling has been unclear. Recently, however, ZBP1/DAI has been suggested to interact with IAV RNA and trigger RIPK3-RIPK1-caspase-8 mediated cell death in BMDMs [24,85] and MEFs [23,144] (Figure 2). These events are regulated by ZBP1′s unique structure, where it contains a Zα domain that binds the nucleic acids, and two RIP homotypic interaction motif (RHIM) domains that allow its association with RHIM-containing proteins, RIPK1 and RIPK3. Similar RHIM–RHIM interactions between TRIF and RIPK3 have been linked to TLR3/4-driven apoptotic caspase-8 and necroptotic MLKL signalling in BMDMs [72,148]. In line with the increased susceptibility of RIPK3-caspase-8 signalling deficient mice to IAV infection, BMDMs lacking RIPK3, RIPK3/caspase-8 or RIPK3/FADD were fully resistant to PR8-induced cell death [85]. Activation of caspase-8 signalling and NLRP3 inflammasome-dependent IL-1β activation were abrogated in *Zbp1^−/−^* BMDMs compared to wild-type BMDMs, defining an upstream role for ZBP1 in mediating RIKP3/caspase-8-dependent apoptosis and NLRP3 activation. Likewise, ZBP1 was shown to recruit and activate RIPK3 in the LET1 murine lung alveolar type I line [23], yet, no defect in necroptosis was observed in ZBP1-deficient cells. Akin to RIPK3 deficiency, a recent study reported that mice lacking ZBP1 were hypersusceptible to PR8 infection with elevated viral loads [23]. However, a further study conversely showed that ZBP1 signalling induces airway inflammation to protect mice and reduce the viral load [90]. Overall, the results suggest that a ZBP1-RIPK3-mediated cell death axis exists to limit viral replication in mice [23,85]. It is important to note that the widely used *Zbp1*^−/−^ mice generated in Shizou Akira’s laboratory have been shown to be on a mixed-genetic C57BL/6 and 129 background, complicating in vivo studies that used C57BL/6 controls, rather than littermates [149]. However, in agreement with the hypersusceptibility observed in vivo, a lack of DAI/ZBP1 in murine LET1 cells resulted in increased PR8 replication in vitro [23]. More recently, IRF1 has been shown to be a transcriptional regulator of DAI/ZBP1 to promote apoptotic caspase-8-mediated cell death and NLRP3 inflammasome activation in BMDMs, and possibly MLKL-mediated necroptosis in murine lung fibroblasts [86]. However, in contrast to the pathology observed in *Zbp1^−/−^* mice, IRF1 knockout mice did not display altered susceptibility or viral loads upon PR8 infection, which was suggested to potentially reflect redundancy or that residual DAI/ZBP1 levels were sufficient for IAV responses in vivo. It is important to note that ZBP1 is an interferon-stimulated gene (ISG) in murine but not human cells [150]. In addition, ZBP1 is evolutionarily absent is avian species [151], which represent the main reservoir of IAV. Together, these lines of evidence suggest that a fundamental difference exists in the role of ZBP1 between species.

## 7. Potential for Apoptotic Caspase-3-Induced Gasdermin E (GSDME)-Mediated Pyroptosis in IAV

As discussed above, GSDMD is cleaved by caspase-1 following NLRP3 inflammasome activation and GSDMD-NT forms a pore in the plasma membrane to facilitate pyroptosis. More recently, it has been shown that related family member gasdermin E (GSDME) can be cleaved between its N- and C-terminal domains (at Asp 270) by caspase-3 during apoptosis [96] (Figure 2). The cytotoxic GSDME N-terminus (GMDME-NT) is released to form a pore on the plasma membrane and causes loss of membrane integrity. It is currently thought that GSDME-dependent pyroptosis does not facilitate IL-1β release [152] and thus, it has been proposed that pyroptosis could be redefined as ‘gasdermin-induced necrotic cell death’ [152]. As GSDME is a downstream target of apoptotic caspase-3, GSDME-mediated necrosis may possibly play a role in promoting inflammation during IAV infection, however, its role has yet to be explored.

## 8. Necrosis and Secondary Necrosis during IAV Infection

Primary or accidental necrosis is defined by loss of plasma membrane integrity and cell membrane rupture, resulting in the release of DAMPs and intracellular organelles. Primary necrosis is thought to generally result due to extreme insults, including viral infection. More recently, secondary necrosis has been identified as a form of programmed cell death and refers to the progressive loss of integrated plasma membrane of apoptotic cells. Secondary necrosis presents with very similar cellular features to pyroptosis but is GSDMD- and GSDME-independent [96,97,153] and differs to primary necrosis with loss of chromatin [154] (Table 1). Apoptotic cells that are not cleared (e.g., by phagocytic cells) may progress to secondary necrosis, in addition to potential GSDME-mediated necrosis. Necrotic pulmonary epithelial cells have been observed in lung tissue sections from fatal human cases of the 1918 [80] and 2009 [81] pandemic H1N1 (Table 2). In addition, necrosis has been reported following in vitro IAV infection of primary human bronchial [78] and alveolar type I-like and type II epithelial cells [79]; however, the induction of primary versus secondary necrosis during IAV infection has not been characterised and the pathways involved are not known.

## 9. Concluding Remarks

Severe IAV infections are associated with hyperinflammation and damage to the lung epithelium, leading to the development of ARDS. While apoptosis is thought to result in minimal inflammation, necroptosis and GSDMD-mediated pyroptosis result in the release of DAMPs, such as IL-1β and HMGB1 (Figure 2), which have the potential to promote a hyperinflammatory response. Inflammatory responses in IAV-infected mice deficient in extrinsic and intrinsic apoptosis, necroptosis and pyroptosis, remain to be fully elucidated. However, a lack of the upstream IAV sensor DAI/ZBP1 restricts RIPK3-RIPK1-caspase-8 signalling-induced cell death and NLRP3 inflammasome activation [85] (Figure 2).

Apoptosis, which traditionally does not allow for the lytic release of inflammatory DAMPs, is thought to play an important role in limiting viral replication, as mice with impaired apoptotic cell death display increased viral loads [23,85,145,146]. Moreover, IAV infection of primary macrophages is largely abortive with the exception of some H5N1 strains, and cell death by apoptosis is thought to block viral replication [7,8,9]. Hence, depletion of macrophages in vivo during IAV infection results in increased lung viral loads [8]. Neutrophils have also been shown to limit viral replication in vivo [57,58], however, the cell death pathways which control their survival are not known. The potential of lytic cell death pathways, such as pyroptosis and necroptosis, to facilitate the release and dissemination of IAV, has also not been examined in detail. In one study, mice lacking MLKL did not display altered viral loads, suggesting that necroptosis does not limit viral replication [145].

Intriguingly, another level of complexity in death receptor-mediated cell death has been observed in a tissue- and cell-specific manner during IAV infection. As previously discussed, TRAIL has been shown to be expressed by PR8-infected alveolar macrophages in vivo and to induce epithelial cell apoptosis [53]. In addition to the significant cell death of bystander cells to IAV-induced death receptor ligands, patients with fatal ARDS-like H7N9 disease have been shown to express significantly less expression of the inhibitor of apoptosis protein, cIAP2, in lung tissue samples [143]. Correspondingly, mice lacking cIAP2 displayed increased susceptibility to PR8 infection, where use of bone-marrow chimeras revealed that this hypersusceptibility was largely attributable to RIPK3-dependent necrosis in the non-haemopoietic compartment, such as bronchial epithelial cells [155]. Bronchiolar epithelial necrotic damage was triggered by hematopoietic production of FasL and TRAIL. The minimal effect of cIAP2 deficiency on the myeloid cell death and inflammatory responses could be explained by a lack of relevant receptors, or the presence of another key cell death inhibitor. It is worth mentioning that XIAP critically represses myeloid cell apoptotic, necroptotic and NLRP3 inflammasome responses in the case of LPS, TNF and Epstein-Barr virus (EBV) infectious responses [72,156,157].

As discussed throughout this review, the IAV proteins NS1, PB1-F2, M1 and NP have been shown to induce apoptosis and NLRP3-mediated pyroptosis (Figure 2). Early induction of cell death would not allow sufficient time for induction of pro-inflammatory responses and viral propagation. The NS1 protein may potentially limit early apoptosis induction by suppressing the production and actions of type I IFNs that inhibits caspase-3 in human epithelial cells [33], as well as induces anti-apoptotic proteins, such as Scribble [133]. Interestingly, a study in human epithelial cells suggested that apoptosis is induced early following IAV infection, but that there is a shift to pro-inflammatory pyroptosis in later stages of infection under the control of IFNs [33].

Overall, the mechanisms involved in balancing the induction and repression of cell death and the interplay with inflammatory pathways requires greater evaluation. Understanding the pathways and mechanisms involved in the induction of immunopathology and fatal disease is imperative, to allow the development of improved and better targeted treatments. The majority of the studies, with the exception of those examining DAI/ZBP1, have examined cell death pathways in epithelial cells. Additional studies which compare responses in different primary cell types, in response to a range of IAV strains at biologically relevant infectious doses, are needed. Lastly, the crosstalk between the different cell death pathways in the context of IAV infection warrants further characterisation.

## Figures and Tables

**Table 1:** Cell death pathways and their features.

Cell Death Pathway	Extrinsic Apoptosis	Intrinsic Apoptosis	GSDMD-Mediated Pyroptosis	GSDME-Mediated Pyroptosis	Necroptosis
Regulation	Regulated	Regulated	Regulated	Regulated	Regulated
Activators	Death receptor and ZBP1 ligation.	IAV PB1-F2, DNA damage and metabolic stress.	ATP, IAV viral RNA, IAV Matrix 2 and PB1-F2 proteins.	Not determined.	Death receptors and TLR ligation.
Morphological features	Plasma membrane blebbing, cell shrinkage, apoptotic bodies, nuclear and DNA fragmentation.	Plasma membrane blebbing, cell shrinkage, apoptotic bodies, nuclear and DNA fragmentation.	Disruption of plasma membrane, cell swelling.	Disruption of plasma membrane, cell swelling.	Disruption of plasma membrane and cell swelling.
Mediating Effectors	RIPK1, RIPK3, FADD and Caspase-8, -3 and -7.	BAX, BAK, APAF-1, cytochrome *c* and Caspase-9, -3 and -7.	NLRP3, Caspase-1, -4, -5, -8, -11 and GSDMD.	Caspase-3 and GSDME.	RIPK1, RIPK3 and MLKL.
Host Inhibitory Molecules	cFLIP, cIAP1/2 and XIAP.	BCL-2 pro-survival family e.g., BCL-2, BCL-XL and XIAP.	C-terminal domain of GSDMD and Caspase 3.	C-terminal domain of GSDME.	cIAP1/2 and Caspase-8.
Key Biochemical Readout	Caspase-8 cleavage.	Caspase-9 cleavage.	GSDMD cleavage.	GSDME cleavage.	MLKL phosphorylation.
Release of cellular contents	No	No	Yes	Yes	Yes
Inflammatory	−/+	−/+	+++	++	++

Apoptotic protease activating factor 1 (APAF-1), Adenosine 5′-triphosphate (ATP), B-cell lymphoma 2 (BCL2), BCL2 associated X protein (BAX), Bcl-2 homologous antagonist/killer (BAK), cellular inhibitor of apoptosis (cIAP), Gasdermin D (GSDMD), Gasdermin E (GSDME), Fas-associated protein with death domain (FADD), Mixed Lineage Kinase Domain Like Pseudokinase (MLKL), receptor-interacting serine/threonine-protein kinase 1 (RIPK1), receptor-interacting serine/threonine-protein kinase 3 (RIPK3), X-linked inhibitor of apoptosis protein (XIAP), influenza A virus (IAV), Z-DNA binding protein-1 (ZBP1), toll-like receptor (TLR) and NOD-, LRR- and pyrin domain-containing protein 3 (NLRP3).

**Table 2:** Cell death pathways identified following IAV infection.

Cell Type	Extrinsic Apoptosis	Intrinsic Apoptosis	Necrosis (Primary or Secondary?)	Necroptosis	Pyroptosis
Airway Epithelial cells	Human primary type I-like alveolar epithelial cells [74].	Human primary type I-like alveolar epithelial cells [74].	Human primary bronchial epithelial cells [78].	Murine primary type I alveolar epithelial cells [82].	Human primary bronchial epithelial cells [33].
	Human lung *adenocarcinoma* A549 type II cell line [75].	Human lung *adenocarcinoma* A549 type II cell line [75,76].	Human primary alveolar type I-like and type II epithelial cells [79].	Murine immortalised LET1 type I alveolar epithelial cells [23].	
	Murine immortalised LET1 type I alveolar epithelial cells [23].	Murine primary tracheal epithelial cells [77].	Epithelial cells in human lung tissue sections [80,81].		
Macrophages and monocytes	Human blood-derived monocytes [83].	Human blood-derived monocytes [83].		Human blood-derived monocytes [83].	Human monocyte-derived macrophages [84].
	Human monocyte-derived macrophages [84].			Murine primary bone-marrow derived macrophages [82,86].	
	Murine primary bone-marrow derived macrophages [24,85,86].				
